# Health economic evaluation of Mirvetuximab soravtansine for the treatment of FRα-positive, platinum resistant ovarian cancer in Germany

**DOI:** 10.1007/s00432-026-06488-8

**Published:** 2026-05-21

**Authors:** Franz F. Janke, Ross J. Baldessarini, Dirk Bauerschlag, Stefanie Zibolka, Michael Hartmann

**Affiliations:** 1 Institute for Hospital Pharmacy, Jena University Hospital, Jena, Germany; 2 International Consortium for Mood and Psychotic Disorder Research, Mailman Research Center, McLean Hospital, Belmont, MA USA; 3 Department of Psychiatry, Harvard Medical School, Boston, MA USA; 4 Department for Gynecology and Reproductive Medicine, Jena University Hospital, Jena, Germany; 5 Central Pharmacy, Magdeburg University Hospital, Magdeburg, Germany

**Keywords:** Cost-effectiveness analysis, Economic evaluation, Health economics, Markov model, Mirvetuximab soravtansine, Platinum-resistant ovarian cancer

## Abstract

**Purpose:**

Mirvetuximab soravtansine (MIRV) is an antibody–drug conjugate targeting the folate receptor α (FRα), approved for FRα-positive platinum-resistant ovarian cancer based on improved progression-free and overall survival and higher response rates compared with standard therapy in the MIRASOL trial. The aim of the present study was to provide a health economic evaluation of MIRV from the perspective of the statutory health insurance in Germany.

**Methods and findings:**

A Markov state-transition model with three health states (stable, progressive, dead) was used with a time horizon of fives years. Transition probabilities were derived from published Kaplan–Meier survival curves of the MIRASOL trial. Direct costs and literature-based utilities were applied. Cost-effectiveness was assessed using the incremental cost-effectiveness ratio (ICER) and the incremental cost-utility ratio (ICUR). Deterministic and probabilistic sensitivity analyses addressed the uncertainty of the model. In the base case, MIRV resulted in an incremental gain of 0.408 life years (LYs) or 0.226 quality-adjusted life years (QALYs) compared to standard therapy. Incremental costs of €128,338.84 were incurred. This resulted in an ICER of €314,753.36 per additional LYs and €567,734.77 per QALY. All analyzed scenarios exceeded the defined threshold, of three times the German gross domestic product per capita, with the drug price having the greatest influence.

**Conclusion:**

MIRV is highly likely to be a more effective treatment option than the chemotherapies currently available for patients with FRα-positive, platinum-resistant ovarian cancer, as approximately 75–90% of ovarian cancers are FRα-positive. However, in the current circumstances, MIRV is unlikely to be considered cost-effective within the German healthcare context without reduction in the drug price.

**Supplementary Information:**

The online version contains supplementary material available at 10.1007/s00432-026-06488-8.

## Introduction

In Germany, around 7,200 women are diagnosed with ovarian cancer each year, corresponding to lifetime risk of approximately 1:74 (DKR 2023; International Agency for Research on Cancer 2024a). Ovarian cancer accounts for about one-third of all malignant neoplasms of the female genital tract but causes half of all deaths from such tumors (DKR 2023). Despite declining incidence and mortality rates, the prognosis remains poor, as 73% of diagnosis are made at an advanced stage (FIGO III/IV) (DKR 2023). The relative 5-year survival rate decreases with advancing stage at diagnosis and is 43% in stage III and only 21% in stage IV (DKR 2023). Mortality is also high globally, which is due, among other things, to late diagnoses and limited access to modern targeted therapies (DKR 2023).

Ovarian cancer is the third most common fatal gynecological tumor disease after breast and cervical cancer (International Agency for Research on Cancer 2024b). After completing first-line therapy, approximately 80% of patients experience a recurrence (Climent et al. 2023). As the disease progresses, many tumors develop resistance to platinum-based chemotherapies, with only about 16% of patients responding to the fifth line of therapy (Kessous et al. 2021). Low response rate highlights the need for innovative approaches. FRα is emerging as a new therapeutic target because it is overexpressed in highly serous epithelial ovarian and endometrial carcinomas. In the SORAYA study, 36% of the patients with platinum-resistant ovarian cancer who were examined showed high expression of FRα (Matulonis et al. 2023).

This therapeutic approach is pursued by the first-in-class antibody–drug conjugate Mirvetuximab soravtansine (MIRV), which specifically targets FRα. MIRV consists of the humanized IgG1 antibody Mirvetuximab, which specifically binds to FRα, and the cytotoxic agent Soravtansin, a maytansinoid derivative (DM4). Both components are held together by a cleavable chemical linker (Moore et al. 2023). After binding to the target cell, the complex is internalized and the cytotoxic component is released to act as a potent tubulin inhibitor, disrupting microtubules, inducing cell cycle arrest in mitosis, and initiating apoptosis.

The efficacy and safety of MIRV were investigated in the randomized, open-label Phase 3, active-controlled MIRASOL study. In this study, MIRV showed significantly prolonged progression-free survival (5.62 vs. 3.98 months), prolonged overall survival (16.46 v. 12.75 months), and a higher response rate (42.3% vs. 15.9%) compared to standard therapy (paclitaxel, topotecan, liposomal pegylated doxorubicin). The adverse event profile also was more favorable, with serious adverse events occurring less frequently with MIRV (42%) than with standard therapy (54%) (Moore et al. 2023). However, new safety signals were observed such as keratopathy (36%) and dry eyes (25%) (Moore et al. 2023). Based on these findings, MIRV was approved in November 2024 by the European Commission (EC) for monotherapy in adult patients with FRα-positive, platinum-resistant high grade serous epithelial ovarian, fallopian tube, or primary peritoneal cancer, who have received one to three prior systemic treatment regimes. (European Medicines Agency^2023^). It was also designated as an “orphan Drug”.

The costs of new drugs, especially for oncology, is rising steadily despite various political measures to limit spending. In Germany alone, spending on patent-protected drugs rose by 18% to around €2.54 billion in the period from February to April 2024 compared to the previous year. Oncology drugs dominated the list of the 40 drug groups with the highest costs. Therefore, the treatment of cancer is a resource-intensive area with in the healthcare system (Ludwig et al. 2024). Given limited financial resources, the question of how to use these expensive treatments efficiently is becoming increasingly urgent (Szucs et al. 2021).

With that in mind, cost-effectiveness analyses (CEAs) are gaining importance, and health economic evaluations should be an integral part of rational allocation decisions, especially in settings where limited resources must be distributed efficiently (Szucs et al. 2021). In the German healthcare system, CEAs may become particularly relevant during the AMNOG (Reform of the Market for Medicinal Product Act) process, which coordinates reimbursement negotiations between pharmaceutical manufacturers and the German statutory health insurance (GKV). If these negotiations fail to reach an agreement on pricing, an arbitration board can mandate a formal cost-effectiveness analysis to inform the final decisions.

It should be noted that without an explicit assessment of the costs and utilities of alternative interventions, it is not possible to set well-fonded priorities. Failing to do so is a decision in itself and carries the risk of inefficient use of limited resources or overlooking more effective therapies (Neumann and Sanders 2017).

This report appears to represent the first health economic evaluation of MIRV in the context of the German healthcare system, although comparable health economic evaluations are currently available for the US and Japanese healthcare systems (Zhu et al. 2024; Miki et al. 2025). The aim of this study was systematically to examine the key factors influencing the cost-effectiveness of MIRV and to evaluate conditions under which the new treatment could be considered as cost-effective from the perspective of GKV in Germany. These results should then be compared with those of the models from the predominantly privately financed US healthcare system and the Japanese healthcare system, which is contribution-based and supported by government subsidies.

## Materials

### Model structure

**Fig. 1:**
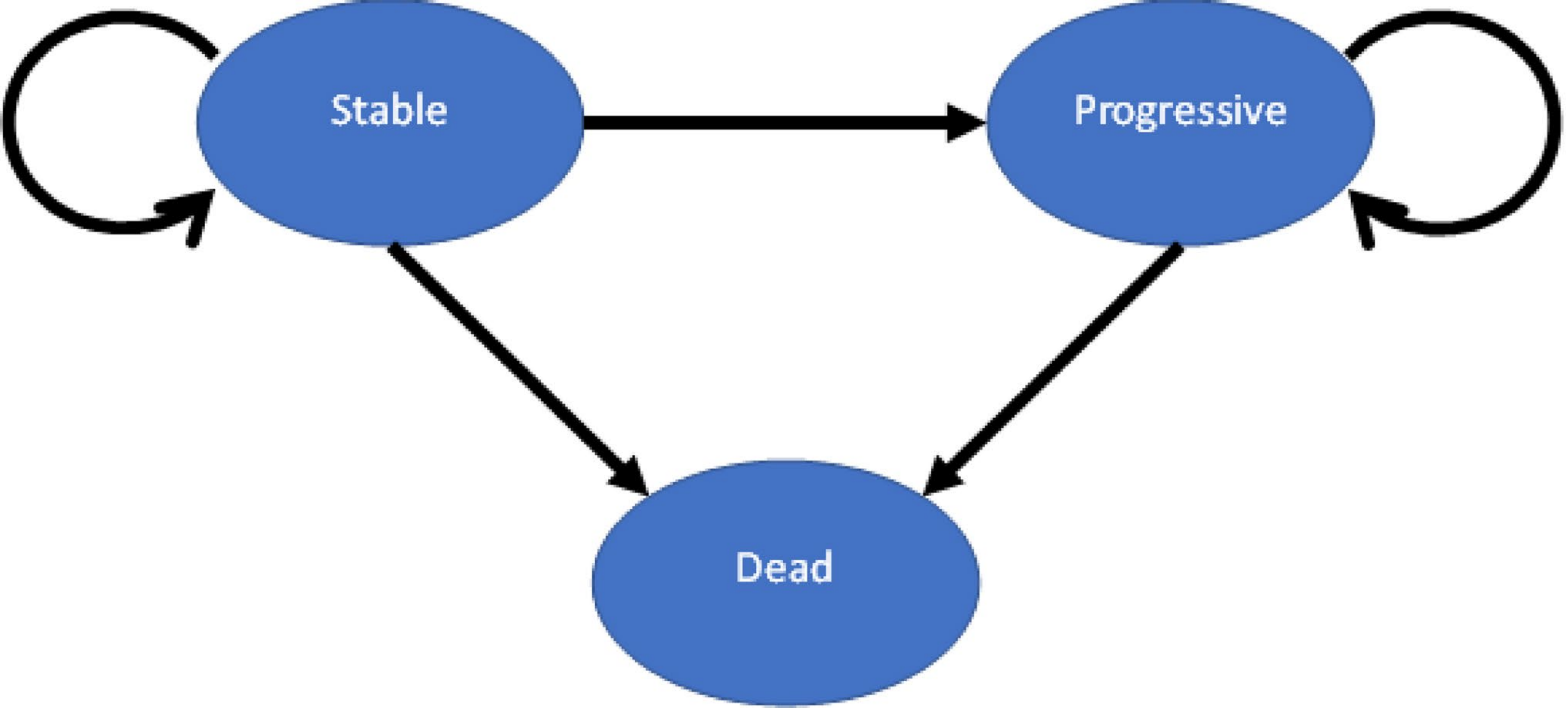
State transition model structure

The state transition model used (Fig. 1) was a Markov model implemented using TreeAge Pro Healthcare 2025 software (version 25.1.1, LLC; Williamstown, MA, USA). The model architecture follows a state transition model comprising three health states: stable, progressive and, dead. All patients were initially in the stable state. From there, they could either remain in that state or transition to the progressive state as defined in the study protocol (Moore et al. 2023). Patients in the progressive state could remain there or die, but return to the stable state was not possible.

The model operated with a cycle length of four weeks. Costs and utilities were discounted at an annual rate of three percent in accordance with recommendations of the Institute for Quality and Efficiency in Health Care (IQWiG) (IQWiG 2023). Modeling the transition probabilities as time-dependent allows for a more realistic representation of disease progression than would be possible with constant values (Sonnenberg and Beck 1993). The probabilities were based on published Kaplan–Meier survival curves from the MIRASOL study (Moore et al. 2023) and were calculated independently of individual patient history. A model time horizon of five years was chosen, which corresponds to a common period in oncological evaluation (Institute for Clinical and Economic Review 2024).

Since the original data from the MIRASOL study covered an observation period of only about 24 months, an extrapolation of the Kaplan–Meier data was necessary to model effects of exposure time.

For the extrapolation, typical parametric functions for modeling survival data were employed, according to reported recommendations (Latimer 2013). We examined exponential, Weibull, Gompertz, log-logistic, and log-normal distribution functions. The selection of the most suitable function was based on the sum of squared residuals, as well as the Akaike Information Criterion (AIC) and the Bayesian Information Criterion (BIC) (Ishak et al. 2013). Additionally, the resulting model curves were evaluated for their realistic representation by consulting a gynecological oncologist. As a result, the log-logistic function proved to be the most appropriate option for the MIRV group, while the Weibull function provided the best fit for the standard therapy group. Detailed information on the mathematical criteria and graphical fit assessment is provided Section 1.1. of the Supplementary Appendix. Transition probabilities were calculated from the published Kaplan–Meier curves. Where no empirical data were available, extrapolated cohort trajectories were used (Sonnenberg and Beck 1993) based on the following:

$$ P = 1 - e^{{ - \frac{{{\mathrm{ln}}\left( y \right)}}{t}}} $$

where *P* represents the survival probability at time *t* and *y* denotes the proportion of survivors at that time. The transition from the progressive state to death was calculated by subtracting the two given cohorts. In addition to transition probabilities, we also considered natural mortality in the model. The underlying mortality rates were based on data from the German Federal Statistical Office, reflecting the median age of the patient population (Statistisches Bundesamt 2024). Detailed calculations of the transition probabilities can be found in the appendix.

### Patient population and treatment options

The patient population considered in the model is based on published data from the MIRASOL study (Moore et al. 2023). The methodological framework is described in the corresponding study protocol. A total of 453 patients participated and, were randomized to receive MIRV monotherapy or standard treatment in a 1:1 ratio. Inclusion criteria included a platinum-resistant, high-grade serous epithelial carcinoma of the ovary, fallopian tube, or peritoneum, as well as high expression of FRα, defined as ≥ 75% of tumor cells showing moderate to strong membrane staining in immunohistochemistry. Additionally, all patients must have received one to three standard treatments already.

In the intervention group, 227 patients received MIRV at a dose of 6 mg per kilogram of adjusted ideal body weight, administered in a three-week cycle. The 226 patients in the comparison group were treated with standard therapy chosen by the treating physician. Standard treatment options included Paclitaxel at a dose of 80 mg/m^2^ on days 1, 8, 15, and 22 of a 28-day cycle, pegylated liposomal Doxorubicin at a dose of 40 mg/m^2^ on day 1 of a four-week cycle, or Topotecan, administered either in a four-week cycle (4 mg/m^2^ on days 1, 8, and 15) or in a 21-day cycle (1.25 mg/m^2^ on days 1 to 5). The choice of the specific regimen was made individually by the treating physician, taking into account clinical circumstances and the framework of the study guidelines (Moore et al. 2023).

### Costs

#### General

Only direct costs were included in the health economic evaluation. These included expenses for drug acquisition, adverse event treatment and monitoring. Cost calculation was conducted from the perspective of the GKV. When hospitalization was required, costs were calculated with the German DRG system (Diagnosis Related Groups). Outpatient costs were determined with the Uniform Value Scale (EBM) (Kassenärztliche Bundesvereinigung 2025). The EBM is a nationwide reimbursement system for outpatient services for medical services and procedures in the outpatient sector of the GKV through a point value system (Kassenärztliche Bundesvereinigung 2025).

The treatment with MIRV was classified as an outpatient service in the model. We determined the DRG costs for clinical management of adverse events requiring hospitalization using the WebGrouper tool from the DRG Research Group (DRG Research Group 2025). The drug costs were based on Appendix 3 of the agreement on price formation for substances and preparations from substances, which pharmacies use to claim reimbursement for cytostatic preparations, starting in the first quarter of 2025 (GKV-Spitzenverband 2025).

#### Drug costs

MIRV was administered every three weeks. The dosage was determined according to the study protocol for an average 64-year-old female patient in Germany. To calculate the ideal adjusted body weight, data from the Federal Statistical Office, as well as the formula specified in the study protocol, were used (Moore et al. 2023; Statistisches Bundesamt 2023). At a price of €30.39 per mg, the average costs amounted to €18,281.66 per four-week treatment interval. This interval was chosen to ensure comparability with conventional chemotherapies, which were administered every four weeks. Additionally, premedication specified in the study protocol were taken into account.

For Topotecan, a four-week treatment cycle was assumed. Distribution of the standard therapy arms included 92 patients given Paclitaxel, 91 given pegylated liposomal Doxorubicin, and 53 given Topotecan. We calculated dosages and resulting costs based on an average body surface area of 2 m^2^, dosages and resulting costs were calculated. The average therapy expenses per treatment interval per patient in the standard therapy arm were calculated to be €1,276.83 (Table 1).

**Table 1:** Overview of drug costs per cycle, monitoring, premedication, and adverse evet costs per cycle, according to the MIRASOL study protocol

Cost type	Cost (€)	
	Mirvetuximab Soravtansine	Standard therapy
Drug	Monthly Cost (€)	Proportion of treatment arm
Mirvetuximab Soravtansine	18,231.66	
Any standard therapy	1,276.83	
Paclitaxel	519.77	0.41
Topotecan	299.43	0.23
Pegylated liposomal Doxorubicin	457.63	0.36
Monitoring (€)	96.96	
Imaging (€)		
Cycles 1 to 9	141.12	
As of cycle 9	70.56	
Premedication	82.06	27.93

Adverse events	Incidence (%)	Costs (€)	Incidence (%)	Costs (€)
Anemia	0.88	0.50	9.29	5.28
Neutropenia	0.88	0.41	15.93	7.41

According to the MIRASOL study protocol, the study medication was administered until the occurrence of one of the following disease progression, intolerable toxicity, withdrawal of consent, death, or early study termination by the sponsor (Moore et al. 2023). Post-progression treatments were not explicitly modelled, as detailed information was not available from the trial publication. In the MIRV group, side effects led to discontinuation in 9.2% of patients, and dose reductions were necessary for 33.9%. In the standard therapy group, the discontinuation rate was 15.9%, and dose reductions were required for 24.2% (Moore et al. 2023). This discontinuation was not accounted for, as no detailed data were available.

#### Costs of treating adverse events

For the model only adverse events of grade 3 or higher according to CTCAE (Institute et al. 2017) were considered. The reported adverse events in both treatment groups were tested for significant differences using the Chi-square test (significance level *p* < 0.05). The standard therapies considered for each adverse event based on current German guidelines (Deutsche Krebsgesellschaft 2020). Since no monthly or annual incidences were reported, costs of adverse events were calculated based on cumulative incidence and calculated over the 60-month time horizon of the model (Table 1). Inpatient treatment costs were derived using relevant DRG codes, assuming an average length of stay of 7 days for neutropenia (DRG-Code Q63B) and 6 days for anemia (DRG-Code Q60C) (DRG Research Group 2025).

#### Monitoring and premedication costs

The monitoring costs are derived from the measurements described in the MIRASOL protocol, laboratory tests, ophthalmological examinations and imaging diagnostics. The assessment was based on the EBM catalog and calculated on a monthly basis (Table 1) (Kassenärztliche Bundesvereinigung 2025). A detailed breakdown of premedication costs is provided in the Supplementary Table S4, while Supplementary Table S5 presents a breakdown of all monitoring costs and their corresponding EBM codes.

#### Threshold

Unlike many other international institutions, IQWiG in Germany does not use a fixed cost-effectiveness threshold, instead following the so-called “efficiency frontier”. This indication is specific and based on existing treatment options (IQWiG^2023^). To facilitate international comparisons, we used an alternative approach. The threshold value was chosen as three times the GDP per capita as commonly used benchmark, resulting in a threshold of €155,499 (Statistisches Bundesamt 2025; Braithwaite et al. 2008). We acknowledge the limitations of this approach. Therefore, results are additionally reported for different GDP per capita thresholds, while three times the GDP per capita is used as the primary benchmark.

### Utilities

The assessment of quality of life in terms of gained utility values, such as QALYs, is a central component of cost-utility analyses in the evaluation of new therapeutic approaches. In the MIRASOL study, the impact of MIRV therapy on quality of life was examined, using the EORTC QLQ-C30 (Garcia et al. 2024). The results indicated an improvement in quality-of-life favoring MIRV therapy (Garcia et al. 2024).

A systematic review concluded that only a limited amount of utility data was available for this patient group. Reported utility values for different health states varied greatly and reflected significant methodological heterogeneity (Al-Dakkak et al. 2014).

With that finding, we decided to adopt the utility values used in a model-based health economic evaluation of maintenance therapy in advanced ovarian cancer and we assumed utility values of 0.61 for the stable disease state and 0.5 for the progressive disease state (Nica et al. 2021; Havrilesky et al. 2009).

### Analysis

#### Primary outcome

The comparison of strategies was based on incremental cost-effectiveness ratio (ICER) and the incremental cost-utility ratio (ICUR), expressed in €/LYs and €/QALYs. The aim of this analysis was to identify key factors influencing the ICUR. Additionally, this study aimed to determine under which conditions the new therapeutic approach would be considered cost-effective for the target population.

We addressed uncertainty of the model using deterministic and probabilistic scenario analyses to assess the robustness of the results against changes in key model parameters.

#### Deterministic sensitivity analyses

In these scenario analyses, we varied various parameters to assess their impact on the ICUR. Drug prices varied by 50%, while the costs for treating adverse events and monitoring costs were each considered to vary by 25% (Hallsson et al. 2023). Drug costs were adjusted to a greater extent due to their higher uncertainty compared to the relatively stable pricing of established procedures for managing side effects and monitoring. Early benefit assessment by AMNOG or agreements between health insurance companies and pharmaceutical companies significantly influence drug prices, whereas the procedures for treating adverse events tend to have relatively stable price (Kassenärztliche Bundesvereinigung 2025; Volk 2018; Tunder and Martschinke 2016). As described in the literature, utility values were varied by 20% (Goeree et al. 2016).

#### Probabilistic sensitivity analyses

The probabilistic sensitivity analyses included the key factors of the model and varied them over 10,000 iterations in a first-order Monte Carlo simulation. In accordance with recommendation of the IQWiG, costs were modeled using gamma distribution (IQWiG 2023). To address the uncertainty of the utility values, a beta distribution was used (Briggs 2000). The parameters of this distribution were calibrated based on assumed utility values. A standard deviation consistent with a 95% confidence interval was derived.

As the parametric survival functions were fitted without access to standard errors or variance information, direct sampling of survival parameters was not possible. Therefore, lognormal multiplicative factors were applied to cycle specific mortality and progression transition probabilities in each iteration (Briggs et al. 2012). These factors had a mean of logs of 0, and a standard deviation of logs of 0.10, corresponding to an approximate ± 20% variation (Yong and Shafie 2018). They were sampled once per simulation run and applied consistently across cycles and treatment arms.

#### Utility analyses

The results of the MIRASOL study showed a significantly lower rate of adverse events in the MIRV cohort compared to standard therapy and may represent an improvement of quality of life. Consequently, we conducted a bivariate analysis of utility values to investigate the impact of higher utility values under MIRV therapy. Comparison of patient-specific quality of life in the MIRASOL study revealed that the reduction in quality of life was overall slower and smaller under MIRV therapy (Garcia et al. 2024). Various aspects, such as physical condition, role functioning, and general fatigue, declined more slowly and to a lesser extent than in the standard therapy cohort. We examined this aspect of utility in two additional analyses with defined reduction in utility values per cycle. Accordingly, simulations were conducted for a slow decline with 0.005 utility values and a rapid decline with 0.01 utility values per cycle (Schwarz et al. 2022).

## Results

The modeled population corresponds to the patients included in the MIRASOL study, as described in the Materials section. Briefly, the study population consisted of patients with platinum-resistant, high-grade serous ovarian cancer, exhibiting high FRα expression, who had received one to three prior lines of systemic therapy (Moore et al. 2023).

### Base case results

Compared to standard therapy, treatment with MIRV resulted in a gain of 0.408 LYs and 0.226 QALYs. Simultaneously, incremental costs of €128,338.84 were accumulated. This resulted in an ICER of €314,753.36 per LYs and €567,734.77 per QALY (Table 2). A detailed breakdown of the cost components aggregated in the base case analysis, along with the corresponding EBM fee codes, is presented in Supplementary Table S5.

**Table 2:** Overview of results from base case and deterministic analyses

Scenario	Utilities				Incremental Utilities (QALY)	ICUR (€)
	Stable		Progressive			
	MIRV	Chemo	MIRV	Chemo		
Base case	0.61		0.5		0.226	567,734.77
BVA	0.732	0.61	0.5		0.300	427,122.02
BVA	0.61	0.6	0.5		0.322	398,304.67
BVA	0.732	0.61	0.6	0.5	0.397	323,571.57
Slow decline in both groups	0.61		0.005 per cycle	0.005 per cycle	0.176	728,584.17
Slow decline in MIRV and fast decline in standard therapy	0.61		0.005 per cycle	0.01 per cycle	0.240	534,481.25

*BVA* bivariate analysis, *ICUR* incremental cost-utility ratio, *QALY* quality adjusted life years, *MIRV* Mirvetuximab Soravtansine, *Chemo* standard chemotherapy

### Sensitivity analyses

#### Deterministic sensitivity analyses

**Fig. 2:**
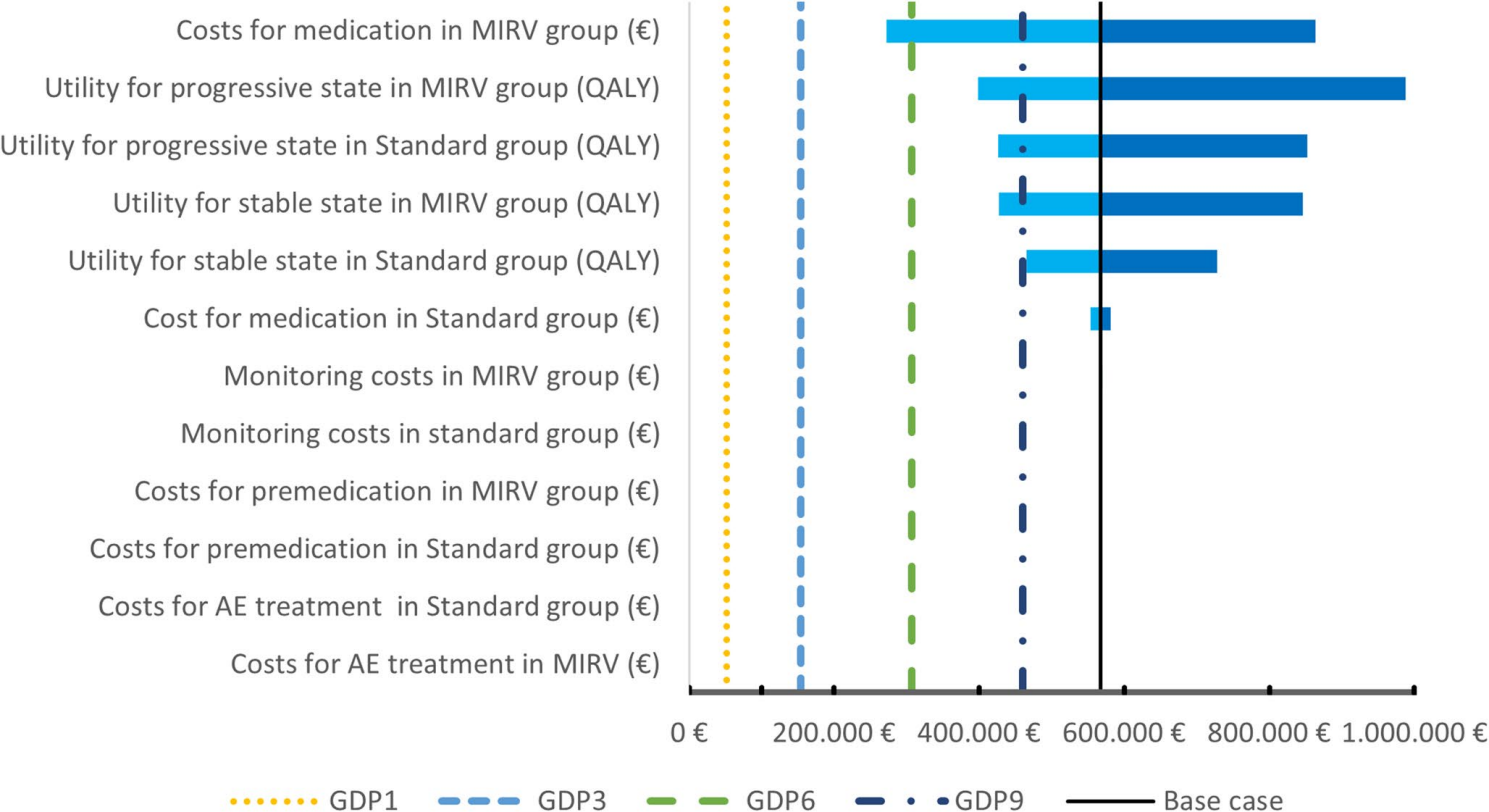
Tornado diagram of the univariate scenario analysis. *AE* adverse event, *QALY* quality-adjusted life years, *GDP* multiples of the gross domestic product per capita

The results of the univariate sensitivity analysis are presented in Fig. 2 as a tornado diagram. Despite the considered parameter uncertainties and the variation of key model assumptions, the ICUR exceeded the established threshold in all scenarios.

We found that the costs for monitoring, adverse event treatment, and premedication had only a relatively minor impact on the result. In contrast, the greatest influence was exerted by the drug costs of MIRV, followed by the assumed utility values of the individual health states.

To further investigate the main influencing factor, we conducted a threshold analysis of the drug acquisition costs of MIRV. It indicated that, at a willingness-to-pay threshold of three times the GDP per capita, total therapy cycle costs would need to remain below €5,550.98. Given the current cycle of €18,231.66 costs, this corresponds to an approximate price reduction of 70%. At a threshold of one time the GDP per capita, the cycle costs would need to be limited to €2,349.57.

We also conducted utility scenario analyses (Table 2). As expected, in bivariate analyses, the ICUR decreased with increasing utility values in the MIRV cohort. However, even with optimistic assumptions, it was not possible to achieve a cost-effective result. For example, increasing the utility values for both the stable and progressive states of the MIRV group by 20% each resulted in an ICUR of €323,571.50, with an incremental gain of 0.397 QALYs. This value still significantly exceeds the chosen threshold. Even under the assumption that the reduction in utility values in the standard therapy occurs twice as quickly, cost-effectiveness with the defined threshold could not be achieved.

Additionally, scenario analyses were conducted using an extended time horizon of 10 years and alternative discount rates of 0% and 5%. These variations had no relevant impact on the cost-effectiveness results, indicating robustness of the model outcomes.

#### Probabilistic sensitivity analysis

**Fig. 3:**
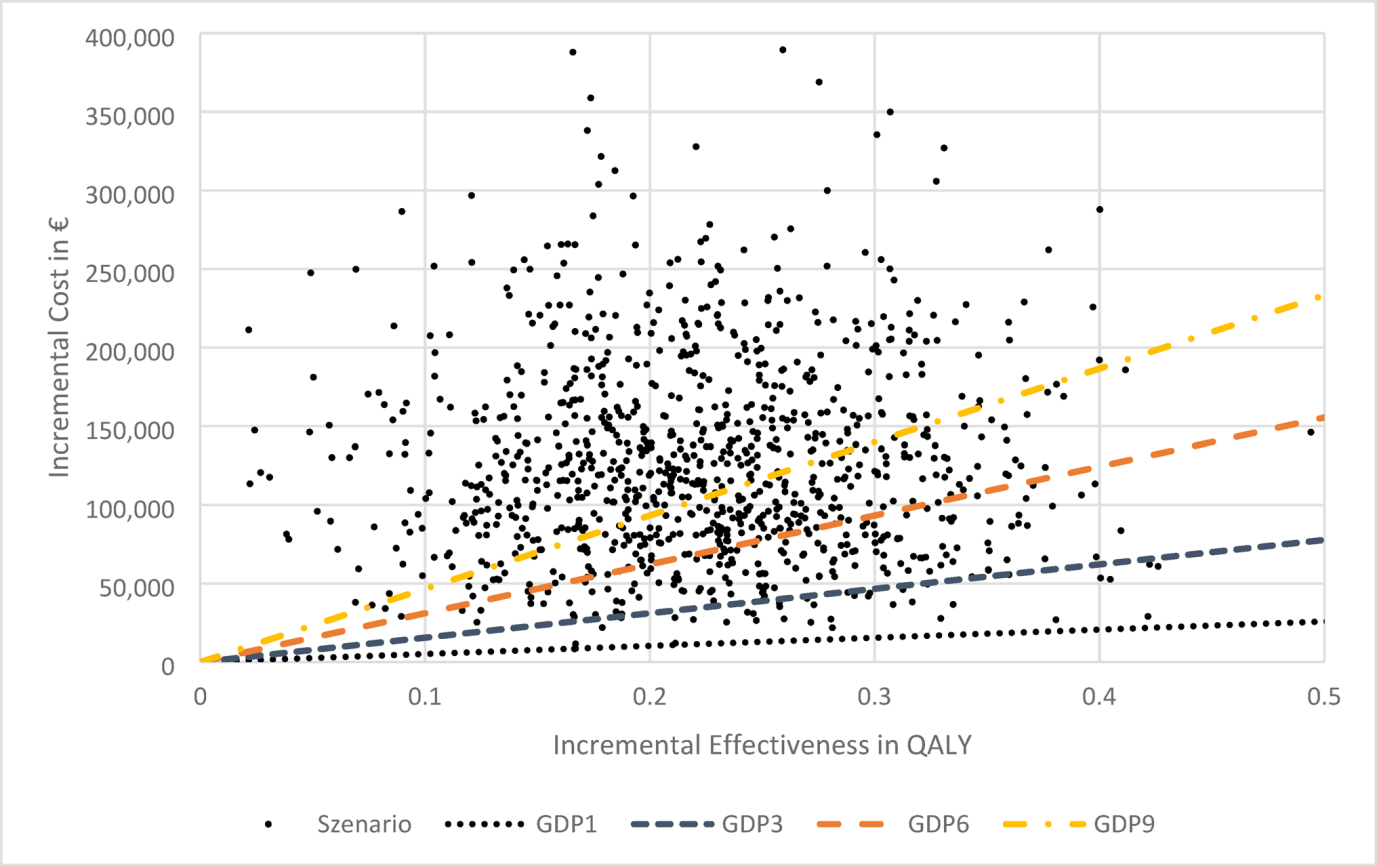
ICUR scatter plot from the probabilistic sensitivity analysis, with cost thresholds indicated as multiples of GDP per capita. *QALY* quality-adjusted life years, *GDP* gross domestic product

An overview of the results from the probabilistic sensitivity analysis is presented as an ICUR scatter plot (Fig. 3). The average ICUR was €679,998 per QALY (95% confidence interval, €642,267 to €717,728 per QALY). Probabilities of achieving cost-effectiveness under variation of the threshold are summarized in Table 3. At a threshold of three times, the GDP per capita, the probability that the intervention could be considered cost-effective is 2.97%.

**Table 3:** Probability of achieving cost-effectiveness at a given threshold

Thresholds based on GDP per capita in € (2024)		Probability of achieving cost-effectiveness threshold in % (QALY)
Factor	Costs [€)	
1	51,833	0.07
3	155,499	2.97
6	310,998	18.45
9	466,497	40.49

*GDP* gross domestic product, *QALY* quality-adjusted life years

## Discussion

There remains a substantial unmet need in platinum-resistant ovarian cancer, as available chemotherapy regimens yield modest response rates and a short duration of benefit (Moore et al. 2023). In Germany, newly authorized drugs with new active ingredients undergo an additional benefit assessment upon market entry (Gemeinsamer Bundesausschuss 2025a). This assessment determines whether the new drug demonstrates added benefit versus the appropriate comparative therapy and forms the basis for pricing and reimbursement negotiations with the GKV. Given the finite financial resources, ensuring the cost-effective use of high-priced therapies is increasingly urgent (Szucs et al. 2021).

All the scenarios conducted demonstrated an increased overall survival and QALYs for the MIRV therapy arm.

In the base case analysis, MIRV was more effective than standard therapies, resulting in an incremental utility value of 0.226 QALYs and 0.408 LYs (Table 2). The resulting ICER was €314,753.36 per LY and €567,734.77 per QALY. These values were significantly above the chosen thresholds, which were aligned with three times the GDP per capita, and did not meet the criterion of cost-effectiveness in the base case.

Even though the base case analysis, as well as the sensitivity and comparison models, presented a consistent picture regarding the high costs and limited cost-effectiveness of MIRV, the significance of the results is limited by several methodologically induced uncertainties.

Within the open label MIRASOL study, there was a trend towards a lower incidence of adverse events in favor of MIRV. Quality of life was assessed using the EORTC-QL30 questionnaire, revealing significantly better scores in global health status, role functioning, and fatigue with MIRV treatment, but response rates were low (Garcia et al. 2024; Gorp et al. 2025). In addition, fewer treatment discontinuations due to adverse events were observed within the MIRV cohort. In the MIRASOL study, 9.2% of patients in the MIRV arm discontinued the therapy due to side effects, compared to 15.9% in the comparison arm. Two analyses concluded that MIRV represents a viable alternative to mono-chemotherapy in patients with FRα-positive, platinum-resistant ovarian cancer (Garcia et al. 2024; Gorp et al. 2025).

A potentially positive impact on quality of life was addressed in deterministic sensitivity analyses. However, even assuming a 20% increase in utility values in both health states of the MIRV therapy, cost-effectiveness could not be achieved. Even with an assumed utility deficit over time under standard therapy there was no substantial effect on the outcome. Furthermore, the quality-of-life data from the MIRASOL trial were not considered for the German benefit assessment due to low response rates and methodological concerns (Gemeinsamer Bundesausschuss 2025b). This indicates that the addressed uncertainty was sufficiently captured with the explored utility ranges.

Throughout the univariate analysis (Fig. 2) the gained utility values did not exert the greatest influence, which instead was primarily driven by the high drug costs. Even a 50% reduction in the costs of acquiring MIRV was insufficient to fall below the chosen cost threshold.

According to these findings, we performed a threshold analysis to determine a price at which MIRV could be considered cost-effective and found that a price reduction of approximately 70% would be required to reach the assumed willingness-to-pay threshold.

Even in the probabilistic sensitivity analysis, the probability of cost-effectiveness remained low under the assumed thresholds. Notably at a threshold equivalent to three times the GDP per capita, the probability of achieving cost-effectiveness was only 2.97%.

An important source of uncertainty arose from the limited availability of health-related utility values for specific treatment options and stages of disease. Since comprehensive utility data for those values were not available, modeling had to rely on literature values, whose transferability to the studied population is only partially assured. The particular relevance of these parameters became evident in the univariate sensitivity analysis, in which the assumed utility values were found among the most influential determinants, ranking second to fourth only to the drug costs for MIRV. We investigated these potential variations within the framework of bivariate scenario analyses.

Moreover, the analyses was based on a state-transition model in which patients could only exist in defined health states. While such models are widely used in health economic studies, they only partially reflect clinical reality. Individual disease trajectories and patient-specific comorbidities cannot be represented. Treatment interruptions or changes in therapy regimens also may be poorly represented. Furthermore, potential state improvements, such as the transition from the progressive to the stable state, could not be accounted for due to limitations in the underlying Kaplan–Meier survival curve data. These curves showed only progression-free survival and overall survival cohorts. The overall survival data included patients who died in both assumed states. In modeling, these circumstances would lead to increased mortality in the progression-free state, with potential underestimates of both costs and gained utility values. These simplifications are inherently model-driven, yet they highlight that health economic modeling always represents an abstracted approximation of complex clinical courses.

External validation of the parametric survival extrapolations using long-term registry data, could further strengthen the robustness of the model. However, such data are typically not stratified by molecular characteristics or prior treatment lines and were therefore not suitable for direct validation in the present analysis. The model therefore relies on the best available clinical trial data for survival estimation.

As previously mentioned, dose reductions or therapy discontinuations were not accounted for in the model, due to the lack of detailed data on these aspects. However, potential cost reductions were addressed within the scenario analysis. In addition, two other health economic evaluations of MIRV reached similar conclusions. One was based on the Japanese statutory health insurance system and used a partitioned survival analysis based on MIRASOL data. In the base case there was an incremental cost increase JPY32,217,876 and a utility increase of 0.281 QALYs, resulting in an ICUR of JPY14,646,442/QALY, which is equivalent to €674,391/QALY. Based on these results, MIRV was not considered cost-effective at the current price, and a substantial price reduction was considered to achieve cost-effectiveness (Miki et al. 2025).

Another evaluation examined the cost-effectiveness of MIRV from the perspective of the American healthcare system. A Markov model with three health states (progression-free, progressed disease, deceased) was conducted, again based on data from the MIRASOL study. In the base case, an incremental gain of 0.90 QALYs with additional costs of $500,574 was determined, resulting in an ICUR of $596,189/QALY, or approximately €553,456/QALY. With an assumed threshold of $100,000/QALY, it was determined that a cost reduction of about 70% would be necessary for MIRV to be considered cost-effective in the American healthcare system (Zhu et al. 2024).

As MIRV is currently approved only for of advanced stages, this therapeutic approach could potentially offer greater benefits if applied in earlier stages of the disease, potentially improving overall survival and quality of life and thereby positively influencing cost-effectiveness (Kong and Zheng 2025; Moore et al. 2021). Nevertheless, regardless of the healthcare system, whether it is the contribution-funded German system, the largely privately financed U.S. system, or the contribution-based, state-subsidized Japanese system, MIRV cannot currently be considered cost-effective. The high drug cost of MIRV is a decisive factor influencing its cost-effectiveness. Accordingly, all evaluations emphasize the need for a substantial price reduction in order for the therapy to be considered cost-effective.

## Conclusion

Although MIRV has clinical advantages over standard chemotherapy, it is not cost-effective as currently priced and a major reduction in drug price would be necessary to meet established cost-effectiveness thresholds.

## Supplementary Information

Below is the link to the electronic supplementary material.

Supplementary Material 1.

## Data Availability

The dataset analysed during the current study are derived from previously published sources (MIRASOL study). Additional datasets generated during the current study are available from the corresponding author upon reasonable request.
