# Supplementary material for: Health economic evaluation of Mirvetuximab soravtansine for the treatment of FRα-positive, platinum resistant ovarian cancer in Germany
Source: J Cancer Res Clin Oncol. 2026 May 21;152(5):111. doi: 10.1007/s00432-026-06488-8 (PMC13194803; doi:10.1007/s00432-026-06488-8)
Supplement: Supplementary file 1 — Supplementary Material 1. [file 432_2026_6488_MOESM1_ESM.docx]

Supplementary Appendix

Supplement to:

Franz F. Janke, Ross J. Baldessarini, Dirk Bauerschlag, Stefanie Zibolka, Michael Hartmann

Health economic evaluation of Mirvetuximab soravtansine for the treatment of FRα-positive, platinum resistant ovarian cancer in Germany

This appendix has been provided by the authors to give readers additional information about the work.

**Health economic evaluation of Mirvetuximab soravtansine for the treatment of FRα-positive, platinum resistant ovarian cancer in Germany**

Contents

[1 Supplementary Materials 2](#_Toc220838722)

[1.1 Extrapolation 2](#_Toc220838723)

[1.2 Monitoring Costs 6](#_Toc220838724)

[1.3 Premedication Costs 7](#_Toc220838725)

[1.4 Base case results 8](#_Toc220838726)

# Supplementary Materials

## Extrapolation

Supplementary Table S1: Results of the extrapolation of Kaplan-Meier survival curves in the MIRV-Cohorts

| **Extrapolation of Kaplan-Meier overall survival curves** | | | | | |
| --- | --- | --- | --- | --- | --- |
|  | Exponential | Weibull | Gompertz | Log-logistic | Log-Normal |
| Residual sum of squares | 0.0321 | 0.0216 | 0.0266 | 0.0206 | 0.0213 |
| Akaike Information Criterion | -191,3391 | -202,9098 | -196,8755 | -204,2610 | -203,2219 |
| Bayesian Information Criterion | -190,6045 | -202,1752 | -196,1409 | -203,5264 | -202,4873 |
| **Extrapolation of Kaplan-Meier progression free survival curves** | | | | | |
|  | Exponential | Weibull | Gompertz | Log-logistic | Log-Normal |
| Residual sum of squares | 0,0205 | 0,0180 | 0,0278 | 0,0091 | 0,0068 |
| Akaike Information Criterion | -204,4418 | -208,1481 | -195,5615 | -227,8861 | -236,4999 |
| Bayesian Information Criterion | -210,4418 | -207,4135 | -194,8269 | -227,1515 | -235,7653 |

Supplementary Figure S1: Results of the extrapolation of Kaplan-Meier overall survival curves in the MIRV cohort

Supplementary Figure S2: Results of the extrapolation of Kaplan-Meier progression-free survival curves in the MIRV cohort

Supplementary Table S2: Results of the extrapolation of Kaplan-Meier survival curves in the standard therapy cohorts

| **Extrapolation of Kaplan-Meier overall survival curves** | | | | | |
| --- | --- | --- | --- | --- | --- |
|  | Exponential | Weibull | Gompertz | Log-logistic | Log-Normal |
| Residual sum of squares | 0,0801 | 0,0144 | 0,0210 | 0,0382 | 0,0331 |
| Akaike Information Criterion | -164,8698 | -214,5977 | -203,7304 | -186,3281 | -190,4591 |
| Bayesian Information Criterion | -164,1352 | -213,8631 | -202,9958 | -185,5935 | -189,7245 |
| **Extrapolation of Kaplan-Meier progression free survival curves** | | | | | |
|  | Exponential | Weibull | Gompertz | Log-logistic | Log-Normal |
| Residual sum of squares | 0,0215 | 0,0158 | 0,0188 | 0,0304 | 0,0211 |
| Akaike Information Criterion | -203,0229 | -211,8655 | -206,9199 | -192,9481 | -203,5371 |
| Bayesian Information Criterion | -202,2883 | -211,1310 | -206,1853 | -192,2136 | -202,8025 |

Supplementary Figure S3: Results of the extrapolation of Kaplan-Meier overall survival curves in the standard therapy cohort

Supplementary Figure S4: Results of the extrapolation of Kaplan-Meier progression free survival curves in the standard therapy cohort

## Monitoring Costs

The derivation of monitoring costs based on the MIRASOL study protocol and is provided in Supplementary Table 1 [1].

Infusion times were derived from the respective summaries of product characteristics. Paclitaxel, pegylated liposomal Doxorubicin, and Mirvetuximab were assumed to require a minimum infusion time of 60 minutes, while Topotecan required 30 minutes [2, 3, 4, 5]. The corresponding administration costs were applied per treatment.

As the MIRASOL study protocol did not specify whether patients received CT or MRI Imagin, it was assumed that 50% of patients underwent CT and 50% MRI. Imaging was applied nine times during the first 36 weeks and every eight weeks afterwards according to the study protocol [1].

Supplementary Table S3: Detailed breakdown of monitoring costs components and corresponding EBM fee codes

| **Procedure** | **EBM code** | **Costs [€]** |
| --- | --- | --- |
| ECOG Performance Status | 03230 | 15.86 |
| **Hematology and clinical chemistry** | | |
| Hematocrit, Hemoglobin, Leukocytes (5-part differential, platelet count) | 32120 | 0.50 |
| Creatinine | 32067 | 0.40 |
| Urea | 32065 | 0.25 |
| ALT | 32070 | 0.25 |
| AST | 32069 | 0.25 |
| Alkaline phosphatase | 32068 | 0.25 |
| Bilirubin | 32059 | 0.40 |
| Glucose | 32057 | 0.25 |
| Albumin | 32435 | 3.13 |
| **Electrolytes** | | |
| Sodium | 32083 | 0.25 |
| Potassium | 32081 | 0.25 |
| Chloride | 32084 | 0.25 |
| Magnesium | 32248 | 1.29 |
| Calcium | 32082 | 0.25 |
| Phosphorus | 32086 | 0,40 |
| Pregnancy test | 32132 | 1,30 |
| CA-125 determination | 32390 | 9,75 |
| **Imaging** | | |
| CT scan of the thorax | 34330 | 72.63 |
| CT scan of the entire abdomen | 34341 | 89.73 |
| MRI of the thorax | 34430 | 130.50 |
| MRI of the abdomen | 34441 | 130.50 |
| Medication administration, infusion min. 10 min | 02100 | 8.30 |
| Medication administration, infusion min. 60 min | 02101 | 20.45 |
| **Ophthalmological examination** | | |
| Visual acuity assessment, slit lamp microscopy, intraocular pressure measurement, indirect fundoscopy | 06212 | 16.86 € |

## Premedication Costs

Recommended premedication was derived from the respective summaries of product characteristics.

For the MIRV cohort, premedication included prednisolone acetate eye drops, lubricating eye drops, intravenous paracetamol, and intravenous dexamethasone prior to infusion [4]. In the chemotherapy cohort, only paclitaxel required premedication, consisting of intravenous cimetidine and intravenous dexamethasone prior to infusion [3]. Costs were calculated in accordance with the German Ordinance on Pharmaceutical Prices (AMPreisV).

The corresponding unit costs and per-cycle cost calculation are presented in Supplementary Table S4.

Supplementary Table S4: Per cycle premedication costs

| **Premedication for MIRV** | **Costs per cycle [€]** |
| --- | --- |
| Prednisolone acetate eye drops | 20.48 |
| Moisturizing eye drops | 3.99 |
| Paracetamol | 40.23 |
| Dexamethasone | 17.37 |
| **Premedication for Paclitaxel** | |
| Dexamethasone | 52.12 |
| Cimetidine | 16.50 |

## Base case results

Supplementary Table S5: Costs and utilities by health state in the base case

|  | **MIRV** | **Standard therapy** |  |
| --- | --- | --- | --- |
|  | **Costs [€]** | **Costs [€]** | **Incremental Costs [€]** |
| MIRV, drug costs | 133,820.42 | - | 127,563.88 |
| Standard therapy, drug costs | - | 6,256.54 | - |
| Adverse Events | 17.16 | 177.23 | -160.07 |
| Monitoring | 1825.36 | 1354.15 | 471.21 |
| Premedication | 600.67 | 136.86 | 463.81 |
| Total | 136,263.61 | 7,924.77 | 128,338.84 |
|  | **LY/QALY** | **LY/QALY** | **Incremental LY/QALY** |
| Stable LY | 0.610 | 0.408 | 0.202 |
| Progressive LY | 0.962 | 0.755 | 0.206 |
| Total LY | 1.572 | 1.164 | 0.408 |
| Stable QALY | 0.481 | 0.378 | 0.103 |
| Progressive QALY | 0.372 | 0.249 | 0.123 |
| Total QALY | 0.853 | 0.627 | 0.226 |

**References**

[1] Moore KN, Angelergues A, Konecny GE, García Y, Banerjee S, Lorusso D, Lee J-Y, Moroney JW, Colombo N, Roszak A, Tromp J, Myers T, Lee J-W, Beiner M, Cosgrove CM, Cibula D, Martin LP, Sabatier R, Buscema J, Estévez-García P, Coffman L, Nicum S, Duska LR, Pignata S, Gálvez F, Wang Y, Method M, Berkenblit A, Bello Roufai D, Van Gorp T. Mirvetuximab Soravtansine in FRα-positive, platinum-resistant ovarian cancer. New England Journal of Medicine 2023;389:2162–74. https://doi.org/10.1056/nejmoa2309169.

[2] Hospira UK Limited. Topotecan Hospira 4 mg/4 ml Konzentrat zur Herstellung einer Infusionslösung – Zusammenfassung der Merkmale des Arzneimittels (SmPC). 2016.

[3] Norton Healthcare Limited. Paxene (INN: paclitaxel) – Zusammenfassung der Merkmale des Arzneimittels (SmPC). n.d.

[4] AbbVie Deutschland GmbH & Co. KG. ELAHERE (INN: Mirvetuximab soravtansine) – Zusammenfassung der Merkmale des Arzneimittels (SmPC). n.d.

[5] Baxter Holding B.V. Caelyx pegylated liposomal 2 mg/ml Konzentrat zur Herstellung einer Infusionslösung – Zusammenfassung der Merkmale des Arzneimittels (SmPC). n.d.
